# Match and training injury epidemiology in elite UK netball: a prospective cohort study over one season

**DOI:** 10.1136/bmjsem-2024-002324

**Published:** 2025-01-04

**Authors:** Sara Horne, Aliah F Shaheen, Bill Baltzopoulos, Laura Hills

**Affiliations:** 1 Department of Life Sciences, Division of Sport, Health and Exercise Sciences, Brunel University of London, London, UK; 2 School of Sport and Exercise Sciences, Faculty of Science, Liverpool John Moores University, Liverpool, UK

**Keywords:** Epidemiology, Netball, Sporting injuries, Ankle, Knee

## Abstract

**Objective:**

To describe the incidence and characteristics of match and training injuries in the UK Vitality Netball Superleague (VNSL).

**Methods:**

Ninety players were observed over one 14-month VNSL season (2021), including pre-, in- and post-season periods. Team physiotherapists recorded injuries using an online surveillance system, classifying them by location, type, mode, mechanism and impact, including severity (time-loss days, TL) and medical attention days (MA). Injury incidence (I) and TL/MA injury burden were calculated per 1000 player hours. χ^2^ analysis compared match and training differences.

**Results:**

Thirty-nine players sustained 70 injuries (n=35 match, 35 training). Match incidence exceeded training (I=41.12 vs 1.10 injuries). Acute injuries were higher in matches (27 vs 17), while overuse injuries were higher in training (18 vs 3; p=0.001). Contact injuries were higher in matches (21 vs 7), and non-contact injuries were higher in training (10 vs 6; p=0.028). Acute ankle ligament injuries in matches caused substantial TL burden (411.7 days lost), while overuse lower leg injuries in training led to high MA burden (13.8). Anterior cruciate ligament (ACL) injuries were infrequent but burdensome (TL 496). The centre position sustained the most injuries (41%).

**Conclusion:**

This study underpinned implementing the first injury surveillance system in the elite UK netball competition, revealing match injury rates ~40 times higher than in training, with distinct injury characteristics. Findings suggest that prevention should target acute lower limb injuries and overuse conditions. Further research should assess the impact of playing with overuse injuries.

**WHAT IS ALREADY KNOWN ON THIS TOPIC**

**WHAT THIS STUDY ADDS**

- This first netball study comparing the profile of match and training injuries found match incidence was considerably higher than training incidence.
- Contact-related, acute ankle ligament injuries had the highest incidence and time-loss burden in matches.
- Non-contact, overuse leg tendinopathies, with a high medical attention burden, were most common in training.
- Knee anterior cruciate ligament injuries were infrequent but caused significant time loss in matches and training.
- The centre position sustained most injuries in matches and training

**HOW THIS STUDY MIGHT AFFECT RESEARCH, PRACTICE OR POLICY**

- Implementing such an injury surveillance system for elite UK netball will enable continuous, systematic injury data collection, enhancing future injury incidence evidence and informing appropriate prevention strategies.
- Prevention strategies should focus on reducing the incidence and impact of acute lower limb injuries.
- Strategies should also prioritise the prevention of overuse injuries, with further investigation required to assess the impact of playing while managing these injuries.
- Further understanding of position-specific injuries could aid tailored prevention programmes.

## Introduction

 Netball, a predominantly female sport with over 20 million participants, is growing in global popularity.^1^ Its fast competitive nature, repeated dynamic movements^2 3^ and restrictive footwork rule generate considerable forces^4^,^6^ and workloads,^7^,^11^ leading to high injury rates of 11.3 to 500.7 injuries/1000 player hours.^12 13^ Lower limb injuries, particularly ankle and knee ligament sprains,^12^,^23^ are common, often due to incorrect landings or player collisions.^15 16 18 20 21 23^ However, the lack of systematic injury surveillance^24 25^ undermines the clarity and usefulness of the evidence base for effective injury prevention.

Current netball injury studies vary widely in designs, definitions, data collection and reporting, with many older studies not reflecting the modern game.^25^,^27^ Only two elite-level studies follow recent methodological guidelines,^28^ assessing the 2019 Netball World Cup^22^ and Australia’s Suncorp Super Netball (SSN) competition,^23^ with only the latter using an ongoing, systematic injury surveillance system.^25^ Both report high ankle and knee injury rates during matches but lack training injury data. Hence, it is unknown if injuries differ between matches and training. Furthermore, no comparative injury data from the UK’s elite Vitality Netball Superleague (VNSL) competition exists. This prospective cohort study uses robust injury surveillance methods to describe the incidence, types, mode, mechanism and impact of match and training injuries during a competitive VNSL season (including preseason, in-season and postseason) lasting 14 months.

## Methodology

### Pilot study and evolution of the Vitality Netball Superleague (VNSL)

### injury surveillance system

Initial injury analysis conducted during the 2019 VNSL prompted developments in the competition’s injury surveillance process. Mandatory injury reporting was introduced using the Performance Data Management System (PDMS), an online athlete health records system developed by the English Institute of Sport.^29^ Injuries were recorded in the PDMS during the 2021 VNSL following the cancellation of the 2020 season due to the COVID-19 pandemic.

### Study design and setting

A prospective cohort design assessed all injuries during the 2021 VNSL season, lasting 14 months. This included preseason preparation (1 September 2020–12 February 2021), in-season (12 February–27 June), and postseason follow-ups (4 months). COVID-19 protocols condensed the competition (in-season) to 20 rounds, two semifinals, third/fourth play-offs and a grand final over 16 weeks. Five weekends included two rounds played over 4 days (double-headers), and matches were reduced from 15-min to 12-min quarters. Injury recording followed the International Olympic Committee consensus statement and Strengthening the Reporting of Observational Studies in Epidemiology Sports Injury and Illness Surveillance guidelines.^28^

### Patient and public involvement

England Netball collaborated with study design and recruitment support. Findings were shared via reports and conference presentations.

### Participants

Nine English teams (n=137 players) participated in the 2021 VNSL. Each squad comprised 14–18 players. England Netball authorised the study, and consent was gained from team gatekeepers and individual players or parents/guardians.

### Equity, diversity and inclusion statement

The study population included female athletes from diverse racial, ethnic, cultural and socioeconomic backgrounds. Our author team varied in gender, ethnicity, culture, and academic level.

### Data collection

Medical attention, time loss (TL), and match and training injuries were diagnosed, treated and recorded on the PDMS by VNSL team physiotherapists. New and subsequent injuries,^30^ excluding pre-existing injuries and exacerbations, were classified using the Orchard Sports Injury Classification System (v14).^31^ Injuries were categorised by mode (acute or overuse), mechanism (contact or non-contact, sudden or gradual onset) and severity (based on four categories of days lost (TL): 0, 1–7, 8–28 or >28 days). Injury impact was further described using medical attention (MA) days, restricted days (RD) and overall duration (RD+TL). Injury frequency by playing position and month was also recorded. Full study definitions are in online supplemental table 1.

### Data analysis

#### Data quality

The quality of the PDMS data recorded was assessed by its completeness and reliability.^28^ Validity could not be determined without a ‘gold-standard’ comparison.^24^ The injury reporting response rate was calculated as a percentage by dividing the number of teams reporting at least one injury on the PDMS system by the number of participating teams. Completeness of injury data was calculated as a percentage by dividing the number of sections completed on the PDMS by the number of expected completed sections.^32^ The reliability of the VNSL team injury reporting was determined from the between-team variability in injury incidence rates and expressed as a percentage coefficient of variation (%CV).^33^

#### Vitality Netball Superleague (VNSL) injury data analysis

Match exposure was calculated as the proportionate player hours per match (consenting players/total players×7 players) × matches played (round matches+semifinals and finals). Training exposure was calculated as consenting players×mean training hours/week×training weeks. Incidence rates were calculated as the number of injuries/exposure hours×1000. The Wilson Score interval with continuity correction was used to calculate 95% CIs for low incidence rates. Injury TL burden was calculated as mean severity (TL per injury) × injury incidence rate.^28 34^ For non-TL injuries, the burden was calculated using mean MA days as the indicator of severity (MA burden).

Descriptive data are presented separately for matches and training, with injury frequency calculated as the percentage (%) of total injuries, excluding missing data. χ^2^ tests examined differences in injury distribution between matches and training. The test was applied when injury categories had at least five observations.^22^ Significance was set at p<0.05, with post hoc tests applying Bonferroni corrections when significance was established. Statistical analyses were conducted in SPSS (V.29.0.1.0 (171), IBM Corp., USA) and followed the CHecklist for statistical Assessment of Medical Papers statement.^35^

## Results

### Study participants

Ninety players from seven English teams agreed to participate in the study (mean age 24.4 years; 95% CI 23.4 to 25.4, mean height 179.3 cm; 95% CI 177. 6 to 181.1). The participants represented 65.7% of the total English team population (n=137) and 83.3% of the seven consenting teams (n=108).

### Data quality

Seven of the nine English teams provided study consent (77.8%), with a 100% injury-reporting response rate and 97.8% data completeness. Four teams provided training exposure data (57.1%), with low between-team variability (11.9% CV percentage). However, match (80.8%) and training (93.6%) incidence rates showed high variability, ranging from 8.2 to 106.9 injuries/1000 hours in matches and 0.2 to 3.1 injuries/1000 hours in training.

### Epidemiology of injury in the Vitality Netball Superleague (VNSL 2021

Seventy injuries (matches n=35; training n=35) occurred in 39 players, representing a period prevalence of 43.3%. Match player hours were 851.18, resulting in an incidence rate of 41.12 injuries/1000 hours (95% CI 29.22 to 57.32), while training hours were 31 888, resulting in an incidence of 1.10 injuries/1000 hours (95% CI 0.76 to 1.54). Consequently, match injuries were 37.4 times more frequent than training injuries (95% CI 23.45 to 59.85).

### Injury mode, mechanism, impact, severity and burden

Acute injuries were more frequent in matches (n=27 (39.7%) vs n=17 (25%)), while incidences due to overuse injuries were higher in training (n=18 (26.5%) vs n=3 (4.4%); p=0.001). Contact injuries dominated in matches (n=21 (30.9%) vs n=7 (10.3%)), whereas non-contact injuries were more frequent in training (n=10 (14.7%) vs n=6 (8.8%); p=0.03). Three match injuries (4.4%) were recurrent. No differences in injury impact (TL, RD, MA and impact duration) were observed (p>0.05). Severe injuries (>28 days) were more frequent in matches (n=6; TL total 585, median 80 days vs n=2; TL total 380, median 190 days) (table 1). Match injury burden was higher, with greater total TL burden (762.5 days lost/1000 hours; 95% CI 727.50 to 794.40) and MA burden (1465.1; 95% CI 1391.03 to 1542.10), compared with training (TL 13.7; 95% CI 5.44 to 31.50 and MA 29.5; 95% CI 20.02 to 42.99).

**Table 1:** Summary of match and training injuries by mode, mechanism, impact and severity

Injury characteristic	Match injuries				Training injuries				P value
	No. of injuries (%)	I (95% CI)	Total days lost	Days lost m (IQR)	No. of injuries (%)	I (95% CI)	Total days lost	Days lost m (IQR)	
Total Injuries	35 (50%)	41.12 (29.22–57.32)	649	47 (15–108)	35 (50%)	1.10 (0.76–1.54)	422	16.5 (5–144)	
Mode									0.001*
Acute	27 (39.7)	31.72 (21.41–46.45)	649	47 (15–108)	17 (25.0)	0.53 (0.32–0.87)	422	16.5 (5–144)	
Overuse	3 (4.4)	†	0	0	18 (26.5)	0.56 (0.35–0.91)	0	0	
Recurrent	3 (4.4)	†	0	0	0	†	0	0	
Mechanism									0.028*
Contact	21 (30.8)	24.67 (15.72–38.12)	321	24 (10–51)	7 (10.3)	0.22 (0.10–0.47)	28	28†	
Non-contact	6 (8.8)	7.05 (2.87–16.09)	328	164†	10 (14.7)	0.31 (0.16–0.60)	394	5 (5–190)	
Gradual onset	2 (2.9)	†	0	0	9 (13.2)	0.28 (0.14–0.56)	0	0	
Sudden onset	1 (1.5)	†	0	0	9 (13.2)	0.28 (0.14–0.56)	0	0	
Impact									
TL	11 (16.7)	12.92 (6.81–23.73)	649	47 (15–108)	6 (9.1)	0.19 (0.08–0.43)	422	17 (5–144)	0.12
RD	12 (18.2)	14.10 (7.66–25.22)	145	5 (2–11)	12 (18.2)	0.38 (0.20–0.68)	200	9 (7-27)	0.852
MA	21 (31.8)	24.67 (15.72–38.12)	1247	18 (6–90)	26 (39.4)	0.82 (0.54–1.21)	941	31 (11–47)	0.331
ID	17 (25.8)	19.97 (12.05–32.46)	794	19 (6–51)	16 (24.2)	0.50 (0.30–0.83)	622	9 (11-34)	0.622
Severity									
0 days	21 (31.8)	24.67 (15.72–38.12)	0	0	28 (42.4)	0.88 (0.59–1.29)	0	0	
1–7 days	2 (3.0)	†	6	3†	3 (4.6)	†	14	5†	
8–28 days	3 (4.6)	†	58	19†	1 (1.5)	†	28	28†	
> 28 days	6 (9.1)	7.05 (2.87–16.09)	585	80 (49–142)	2 (3.0)	†	380	190†	

Number of injuries and (%): frequency of total injuries, excluding missing data.I: incidence per player-hours. CI: Confidence Intervals.

Days lost = total days., m: median and : interquartile range.

Mode/mechanism missing data: match n=2;. Impact/severity missing data: match n=3, training n=1. 3Three recurrent match injuries are reported separately, and included in injury frequency calculations. Chi-squareχ2 tests exclude missing and recurrent data.

* Significant differences between activity (matches and training) and mode; activity and mechanism.

† Number too small to calculate incidence and IQR, only reported for values n≥5 or n≥4, respectively.

Iincidence per 1000 player-hoursIDimpact duration = time-loss days + restricted daysMAmedical attentionRDrestricted daysTLtime loss

### Body region, body area and clinical diagnosis

The lower limb sustained the most injuries in matches (n=27; 38.6%) and training (n=25; 35.7%). In matches, ankle lateral ligament sprains were most common (n=15; 21%), including four severe injuries (>28 days). Lower leg tendinopathy predominated in training (n=10; 14.3%), primarily affecting the Achilles tendon (n=5; 50%), but without TL. Knee injuries were second most common in matches (n=4; 5.7%) and third in training (n=5; 7.1%), including two anterior cruciate ligament (ACL) injuries causing >28 TL. Five concussions were reported (matches n=2, training n=3), with the two match concussions leading to 8–28 TL days (table 2). Ligament/joint capsule injuries were most common in matches (n=16, 22.9%), while muscle/tendon injuries dominated in training (n=18, 25.7%) (see online supplemental table 2).

**Table 2:** Distribution of match and training injuries by body region, area, clinical diagnosis and severity

Body region body area pathology type	Match injuries						Training injuries					
	No. of injuries (%)	I (95% CI)	0 days lost	1–7 days lost	8–28 days lost	>28 days lost	No. of injuries (%)	I (95% CI)	0 days lost	1–7 days lost	8–28 days lost	>28 days lost
Head and neck	2 (2.9)	*	0	0	2	0	4 (5.7)	*	4	0	0	0
Head	2 (2.9)	*	0	0	2	0	4 (5.7)	*	4	0	0	0
Brain/spinal cord	2 (2.9)	*	0	0	2	0	3 (4.3)	*	3	0	0	0
Contusion	0						1 (1.4)	*	1	0	0	0
Upper limb	3 (4.3)	*	2	0	0	1	3 (4.3)	*	3	0	0	0
Shoulder	1 (1.4)	*	1	0	0	0	2 (2.9)	*	2	0	0	0
Synovitis/capsulitis	1 (1.4)	*	1	0	0	0	1 (1.4)	*	1	0	0	0
Contusion	0						1 (1.4)	*	1	0	0	0
Forearm	1 (1.4)	*	0	0	0	1	0					
Fracture	1 (1.4)	*	0	0	0	1	0					
Hand	1 (1.4)	*	1	0	0	0	1 (1.4)	*	1	0	0	0
Fracture	1 (1.4)	*	1	0	0	0	0					
Synovitis/capsulitis	0						1 (1.43)	*	1	0	0	0
Trunk	3 (4.3)	*	3	0	0	0	3 (4.3)	*	2	1	0	0
Chest	0						1 (1.4)	*	0	1	0	0
Muscle injury	0						1 (1.4)	*	0	1	0	0
Thoracic spine	0						1 (1.4)	*	1	0	0	0
Joint sprain	0						1 (1.4)	*	1	0	0	0
Lumbar spine	3 (4.3)	*	3	0	0	0	1 (1.4)	*	1	0	0	0
Nerve injury	1 (1.4)	*	1	0	0	0	0					
Arthritis	2 (2.9)	*	2	0	0	0	1 (1.4)	*	1	0	0	0
Lower limb	27 (38.6)	31.72 (21.41–46.45)	16	2	1	5	25 (35.7)	0.78 (0.52–1.17)	19	2	1	2
Hip/groin	1 (1.4)	*	1	0	0	0	0					
Abrasion	1 (1.4)	*	1	0	0	0	0					
Thigh	4 (5.7)	*	4	0	0	0	1 (1.4)	*	0	1	0	0
Muscle injury	1 (1.4)	*	1	0	0	0	1 (1.4)	*	0	1	0	0
Muscle contusion	2 (2.9)	*	2	0	0	0	0					
Vascular trauma	1 (1.4)	*	1	0	0	0	0					
Knee	4 (5.7)	*	3	0	0	1	5 (7.1)	0.16 (0.06–0.39)	3	1	0	1
Laceration	1 (1.4)	*	1	0	0	0	0					
Tendinopathy	1 (1.4)	*	1	0	0	0	3 (4.3)	*	3	0	0	0
Cartilage injury	1 (1.4)	*	1	0	0	0	0					
Synovitis/capsulitis	0						1 (1.4)	*	0	1	0	0
Joint sprain	1 (1.4)	*	0	0	0	1	1 (1.4)	*	0	0	0	1
Lower leg	2 (2.9)	*	2	0	0	0	10 (14.3)	0.31	10	0	0	0
Tendinopathy	2 (2.9)	*	2	0	0	0	10 (14.3)	(0.16–0.59)	10	0	0	0
Ankle	15 (21.4)	17.62	5	2	1	4	6 (8.6)	0.19 (0.08–0.43)	3	0	1	1
Joint sprain	15 (21.4)	(10.26–29.59)	5	2	1	4	4 (5.7)	*	1	0	1	1
Tendinopathy	0						1 (1.4)	*	1	0	0	0
Muscle contusion	0						1 (1.4)	*	1	0	0	0
Foot	1 (1.4)	*	1	0	0	0	3 (4.3)	*	3	0	0	0
Bone	0						2 (3.0)	*	2	0	0	0
Tendinopathy	0						1 (1.4)	*	1	0	0	0
Abrasion	1 (1.4)	*	1	0	0	0	0					

Number of injuries and (%): frequency of total injuries.

Severity scores are the number of injuries in each category (0, 1–7, 8–28 or >28 days lost).

Lower limb severity data missing: match n=3, training n=1.

* Number too small to calculate incidence. Incidence and 95% CI only reported for values n≥5.

Iincidence per 1000 player-hours

**Figure 1:**
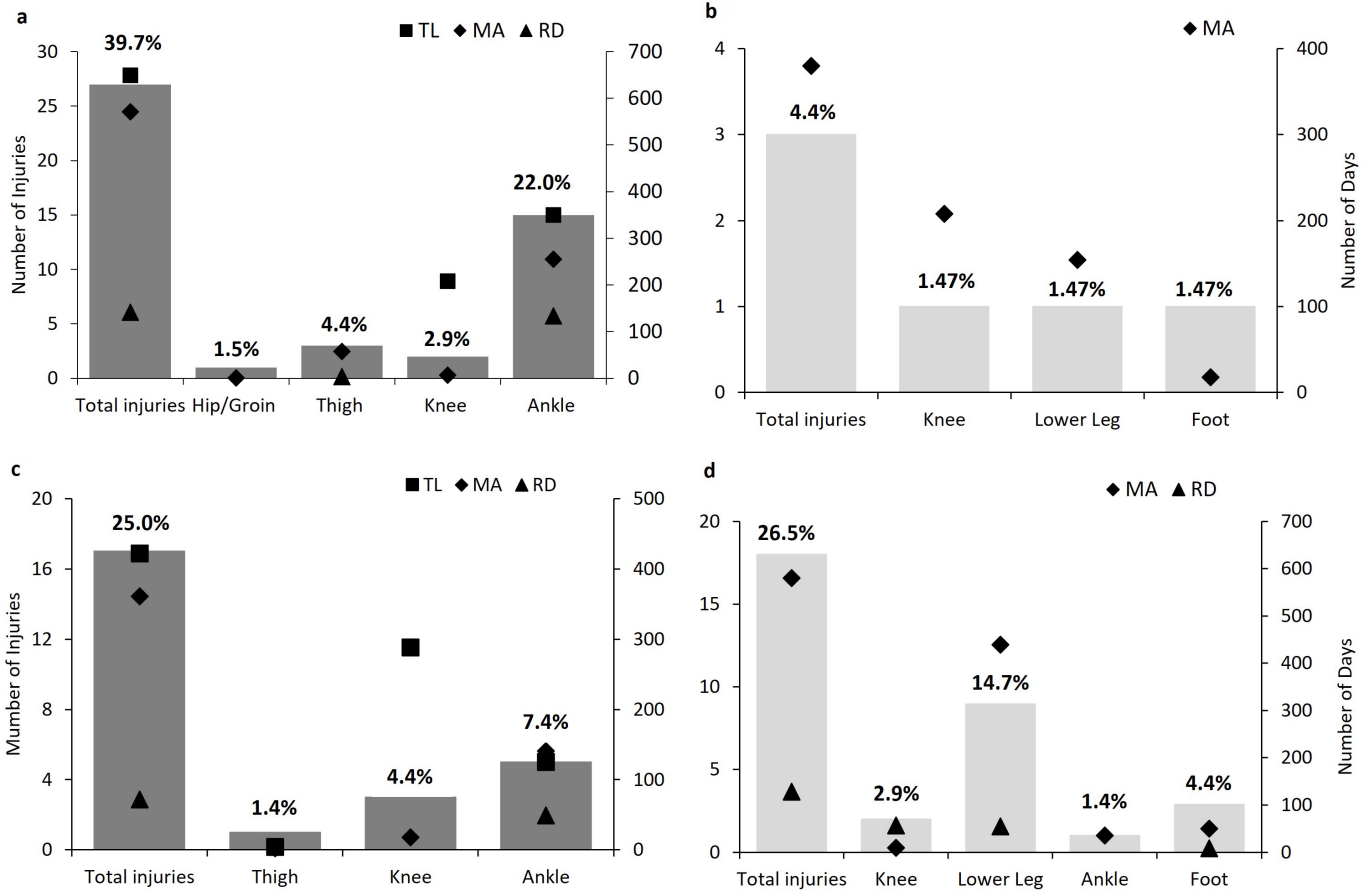
Number of lower limb match (**a, b**) and training (**c, d**) injuries by injury mode (acute a and c, overuse b and d) and impact, compared with total injuries in each category. % values are the frequency of total injuries, excluding missing data. Injury impact values are total time-loss (TL) days, medical attention (MA) days and restricted days (RD). The graph does not represent two recurrent match injuries (n=1 knee, MA=50 days; n=1 lower leg, MA=184, RD=3 days).

Figure 1 shows the mode and impact of lower limb injuries, in relation to the total injuries in each category, for matches and training. All match ankle injuries were acute, mostly contact-related (n=10; 66.7%), causing considerable TL (350 days; median 47 IQR 5–108).

**Figure 2:**
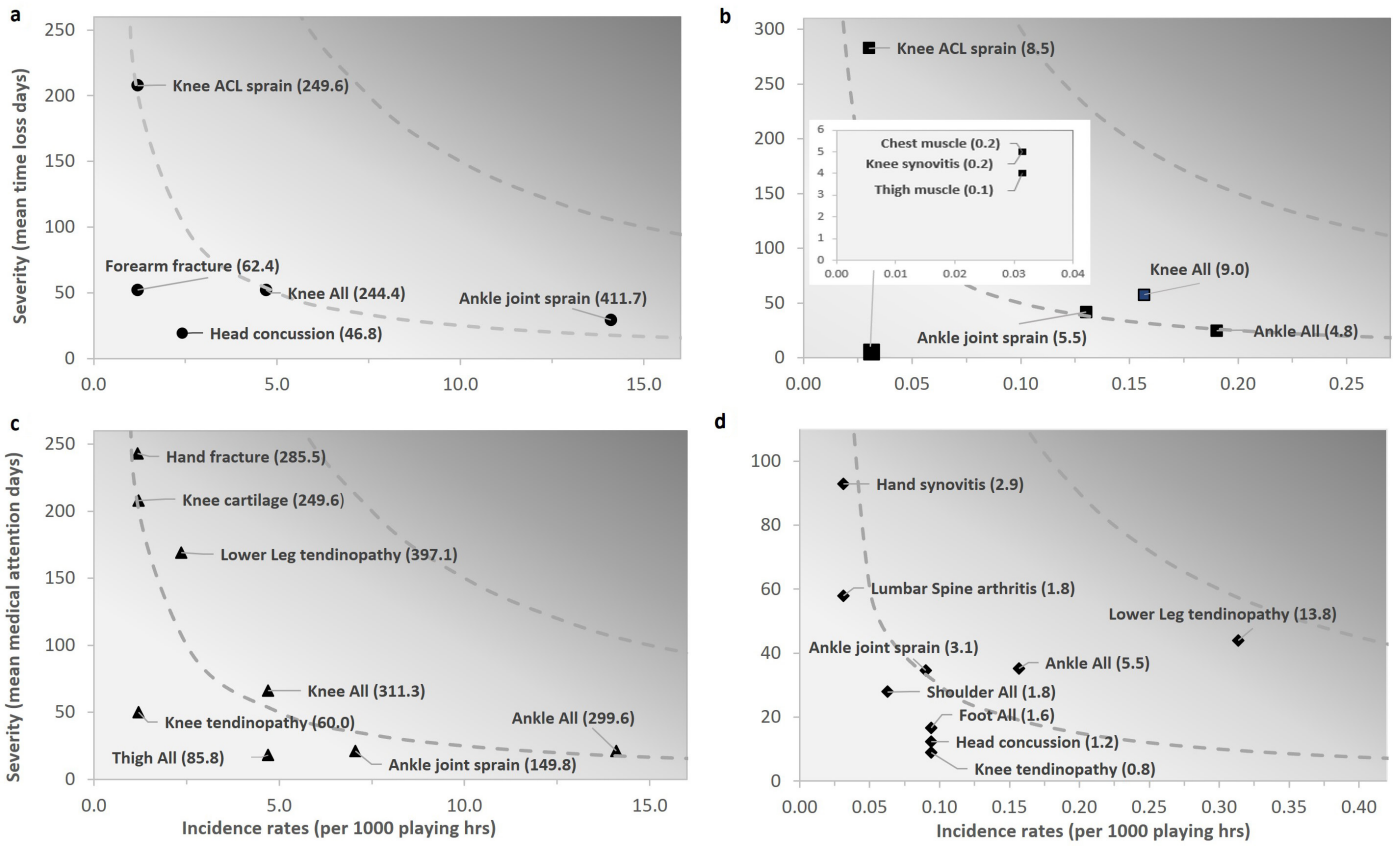
Risk matrix of body area and pathology type burden. Severity is based on the time-loss days for (a) matches and (b) training and medical attention days for (c) matches and (d) training. The darker the grey, the greater the burden. The dotted grey lines represent points of equal burden. ACL, anterior cruciate ligament.

Training lower leg injuries were all overuse, resulting in no TL but substantial MA (439 days; median 30 IQR 22–71). A range of acute and overuse knee injuries were observed, including two acute, non-contact ACL injuries (match n=1; TL 208 days; training n=1; TL 288). Online supplemental table 3 details the impact of injuries by body region, area and diagnosis. The highest TL burden in matches resulted from ankle joint sprains (411.7 days lost/1000 hours) and ACL sprains (249.6), while knee injuries posed the highest TL burden in training (9.0) (figure 2a,b). Lower leg tendinopathy caused the highest MA burden in training (13.8) and matches (397.1) (figure 2c,d).

### Injury by playing position and competition structure

Table 3 shows that the centre position (C) sustained the highest proportion of injuries in matches (n=15; 21.4%) and training (n=14; 20%), resulting in considerable impact in matches (TL 522 days, RD 112 and MA 507) and MA in training (518 days). Ankle (matches n=6; training n=3), lower leg (match n=1; training n=5) and head injuries (match n=2; training n=2) were most prevalent in C, while wing defence experienced the most knee injuries (matches n=3; training n=2).

**Table 3:** Frequency and impact of match and training injuries by playing position

Playing position	Match injuries					Training injuries				
	No. of injuries (%)	I (95% CI)	TL	RD	MA	No. of injuries (%)	I (95% CI)	TL	RD	MA
Goal shooter	3 (4.3)	*	52	0	3	2 (2.9)	*	0	13	156
Goal attack	2 (2.9)	*	5	7	146	1 (1.4)	*	0	57	0
Wing attack	1 (1.4)	*	0	0	15	1 (1.4)	*	0	8	0
Centre	15 (21.4)	17.62 (10.26–29.59)	522	112	507	14 (20.0)	0.44 (0.25–0.76)	4	17	518
Wing defence	8 (11.4)	9.40 (4.38–19.21)	1	14	492	8 (11.4)	0.25 (0.12–0.51)	10	13	127
Goal defence	5 (7.1)	5.87 (2.16–14.48)	69	12	84	6 (8.6)	0.19 (0.08–0.43)	283	32	68
Goal keeper	1 (1.4)	*	0	0	0	3 (4.3)	*	125	60	72

Number of injuries and (%) frequency of total injuries.I: incidence per player-hours. CI: Confidence Intervals.

Injury Iimpact values are total time-loss (TL) days (), restricted days (RD) and medical attention (MA) days.

* Number too small. Incidence and 95% CI were only reported for values n≥5.

Iincidence per 1000 player-hours

**Figure 3:**
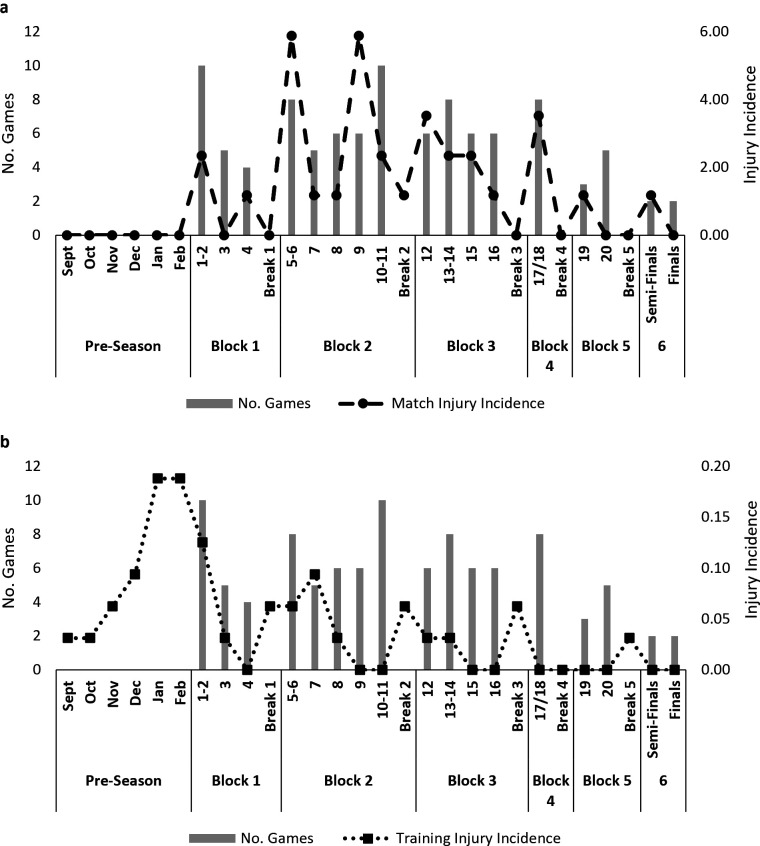
Incidence of match (**a**) and training (**b**) injuries in relation to competition structure: preseason training, number of games each round and across each block of games. Block 1=12 February–1 March, break 1=10 days; block 2=12 March–12 April, break 2=12 days; block 3=25 April–17 May, break 3=10 days; block 4=28–31 May, break 4=12 days; block 5=13–21 June, block 6 (6)=26–27 June.

Nineteen injuries (27.1%) occurred in preseason (63% in the final 2 months), while 51 (72.9%) occurred during the competition. In-season match injuries peaked during block 2, in rounds 5–6 and 9 (n=5; 7%), coinciding with the busiest match period (35 games in seven rounds) (figure 3a). Training injuries decreased post preseason but also increased following double-header weekends in rounds 5–6 and 10–11 and the end of block 3 (figure 3b).

## Discussion

This study prompted the implementing of a systematic injury surveillance system in the VNSL, with the findings representing the first prospective assessment of injuries in elite UK netball over a whole preparation and competition season. It also provides the first comprehensive evaluation of match and training injuries in netball. Furthermore, it is one of the few netball studies to report data quality and introduces a novel method for reporting non-time loss injury burden. The principal findings were that match injury incidence was nearly 40 times higher than training incidence. Most injuries occurred in the lower limb, and there were differences in the injury type, mode, mechanism, impact and burden between matches and training.

### Data quality

Injury recording using the PDMS had a high response rate and data completeness ≥98%. Training exposure calculations based on average team exposures were also relatively accurate, with low team variability (12% CV). However, considerable variation in injury reporting across teams (match 81% CV; training 94% CV) suggests inconsistent interpretation of injury definitions and potential under-reporting,^32^ consistent with previous prospective surveillance studies.^32 36 37^ The limited time available for part-time physiotherapists to record injuries remains a challenge. Promoting accurate reporting and regular PDMS training is recommended to improve future data quality.

### Match and training injuries in the Vitality Netball Superleague (VNSL 2021 competition

Match injury incidence in this study (41.12/1000 hours) was considerably higher than training (1.10/1000 hours), consistent with trends in elite women’s team sports, where match injuries exceed training by 6 to 13 times.^38^,^40^ Training rates were ~3 times lower than soccer (3–3.5)^39 40^ but comparable to women’s rugby (1.5).^38^ However, match rates were 1.7–2 times higher than soccer (19.2–19.6),^39 40^ basketball (24.9)^41^ and rugby-15s (19.6), but 1.5 times lower than rugby-7s (62.5).^38^ Recent netball studies report tournament match rates of 89.4 in sub-elite and 54.76 in elite players,^21 22^ supporting our findings of higher match rates and aligning with research suggesting that tournament rates are higher than season-long rates.^13^ These findings highlight the elevated injury risk during netball matches due to the increased physical demands.

Unlike professional basketball research, which found no differences in injury characteristics between matches and training,^42^ our study revealed clear distinctions. Acute injuries were more common in matches (40%), often due to player contact (31%), aligning with the 2019 World Cup findings.^22^ Conversely, overuse injuries were more frequent in training (27%), typically from non-contact mechanisms (15%), with a similar rate of acute injuries (25%). While the overall impact of match and training injuries was comparable (TL, RD, MA, ID), match injuries were more severe, leading to greater total TL (>28 days: 9%; 585 days). In contrast, training injuries had a higher median TL in the most severe cases (>28 days: 190 days).

The type of injury and their subsequent impact and burden also differed between matches and training. While the lower limb was the most frequently injured body region in both matches (39%) and training (36%), acute lateral ligament ankle sprains, often from athlete contact (67%), led to the highest severity (TL: 350 days and TL injury burden (411 days lost/1000 hours) in matches. In contrast, overuse tendinopathies, typically at the Achilles tendon (30%), resulting in no TL but high MA (439 days) and MA injury burden (14 days/1000 hours), were most common in training. As previously reported,^13 20^ knee injuries were less frequent, but included two notable acute, non-contact ACL cases (match 1; training 1), causing considerable severity (match 208 days; training 288) and TL burden (match 244; training 9 days/1000 hours).

Our match injury findings align with previous netball research,^12^,^23^ consistently identifying ankle and knee injuries as the most frequent and severe. These injuries impact player performance and team success.^43^ Hence, we support Toohey *et al*’s^23^ recommendation that prioritising acute lower limb injury reduction may be netball’s most effective strategy for overall injury control. Additionally, our training injury data highlight a need to concurrently address overuse lower leg injuries due to the high MA burden they place on physiotherapists. Moreover, the effect of playing while undergoing treatment for overuse injuries is not well understood. Evidence suggests that non-TL injuries may precede TL injuries,^44 45^ emphasising the need to explore how playing with overuse injuries influences the risk of subsequent injuries in netball to guide return-to-play strategies.

This study also noted increased player-contact concussion injuries (match n=2, training n=4). While concussion data in netball is limited,^25 26^ recent research identified them as the second most common injury in sub-elite netball,^21^ warranting further investigation into a potential trend. The high overall rate of contact injuries (64%) suggests the increasing physicality of the elite game^27^ is a concern for injuries. In-season injuries (73%) were notably higher than pre-season injuries (27%), contrasting with the 49% preseason rate reported in Australia’s SSN.^23^ While minimising injuries across all phases of competition is important, increasing rates reported during dense match periods requires a particular prevention focus. These findings highlight the need to enhance player resilience through appropriate conditioning programmes and effectively monitor and manage player workloads throughout the season. A greater understanding of the activities leading to injuries, particularly contact injury mechanisms, is important to develop effective training programmes.

To date, injury findings across playing positions have been inconsistent. In this study, the C position sustained the highest injury rates (matches 21%, training 20%), and TL (matches 507 days, training 69 days), consistent with the 2019 Netball World Cup findings^22^ and reflective of the position’s extensive court coverage^2 8^ and versatile role. Conversely, we observed the highest overuse knee injuries (n=4, 6%) without TL in the WD position. Further research is needed to understand potential positional injury differences to aid in developing position-specific conditioning programmes.

### Research implications

Reducing netball injuries benefits player health and team performance and minimises financial costs. Our study highlights the importance of reporting match and training injuries to understand the impact of all injuries in competitive settings. Calculating MA burden highlights the strain non-TL injuries place on team resources and should be included in future studies. Accurate reporting of subsequent injuries, using models such as Hamilton *et al*,^30^ is crucial to understand the relationship between non-TL and TL injuries. Ongoing data collection using this surveillance system will enhance knowledge of injury patterns and inform prevention strategies in elite netball.

### Strengths and limitations

The new VNSL injury surveillance system provided valuable elite-level UK data, enabling the calculation of MA and TL burdens. Modifications to the 2021 VNSL competition may have impacted injury rates, highlighting the need for comparative data from a typical 20-week season to provide further insights into injury trends in this elite competition. Limitations also included inconsistent injury reporting across teams and reliance on average training exposure. Using individual player data from workload tracking devices could improve accuracy and link specific training activities to injuries. Combining match video analysis with input from players and physiotherapists could improve understanding of injury mechanisms.

## Conclusions

The VNSL injury surveillance system revealed that match injuries were ~40 times more frequent than training injuries. Acute, contact-related ankle and knee injuries were prevalent in matches, imposing high TL burden, while non-contact, overuse lower leg injuries, causing high MA burden, predominated in training. Prevention should prioritise acute lower limb injuries while addressing overuse conditions. Further research should investigate the impact of overuse injuries on subsequent injuries and positional injury differences.

## supplementary material

10.1136/bmjsem-2024-002324 online supplemental file 1

## Data Availability

Data are available upon reasonable request.
