# Supplementary material for: Match and training injury epidemiology in elite UK netball: a prospective cohort study over one season
Source: BMJ Open Sport Exerc Med. 2025 Jan 4;11(1):e002324. doi: 10.1136/bmjsem-2024-002324 (PMC11781087; doi:10.1136/bmjsem-2024-002324)
Supplement: online supplemental file 1 [file bmjsem-11-1-s001.pdf]

Supplementary Table 1: Operational definitions used in the collection of 2021 VNSL injury data

| **Operational Definitions** | |
|---|---|
| Injury | Injury resulting from training or match-play during the VNSL season leading to one of the following consequences: 1) medical attention or advice (from team Physiotherapist and/or other medical professional), 2) time-loss (a reduction in the amount or level of netball activity). |
| Match | All competitive matches during the 2021 VNSL competition. |
| Training | Any physical activities under the control or guidance of the 2021 VNSL team's coaching or fitness staff aimed at maintaining or improving players' netball skills or physical condition. |
| First Index Injury | The first injury that occurred during the 2021 VNSL competition. |
| Subsequent Injury | Any injury occurring after the first index injury during the VNSL competition. |
| Types of subsequent Injury [29] | Subsequent New Injury: completely different location to Index Injury |
| | Local Injury: same location but different type to Index Injury |
| | Recurrent Injury (exacerbation): same site and type as index injury but Index injury not fully healed at time of recurrence |
| | Recurrent Injury (reinjury): same site and type as index injury, Index injury fully healed at time of recurrence. |
| Acute Injury | Injury with sudden onset, resulting from identifiable, traumatic event. |
| Contact Injury | Acute injury resulting from contact with another player, or object. |
| Non-Contact Trauma | Acute injury not resulting from contact with another player or object. |
| Overuse Injury | Injury with insidious onset and no known trauma. |
| Sudden Onset | Injury resulting from a specific identifiable event. |
| Gradual Onset | Injury without a specific identifiable event. |
| Injury Severity | Time-loss injuries: the total number of days lost from training and matches recorded from the date of the injury to return to full participation. 0 days lost equates to full participation the day following injury. |
| Severity Categories | 0 days; 1-7 days; 8-28 days; >28 days |
| Healed Injury | A fully healed injury is one where the player has returned to full participation and/or is no longer receiving medical treatment. |
| Medical Attention Days | Number of days players received ongoing treatment with no impact on training or playing. |
| Restricted Days | Number of days players were restricted from training and/or playing |
| Unavailable Days | Number of days players could not participate in training or playing |
| Impact Duration | Number of restricted days + Number of unavailable days |

Supplementary Table 2: Match and training injuries by tissue and pathology

| Tissue Type<br>Pathology Type | Match Injuries | | Training Injuries | |
|---|---|---|---|---|
| | **n (%)** | **I<br>(95% CI)** | **n (%)** | **I<br>(95% CI)** |
| **Bone** | | | | |
| All | 2 (2.9) | * | 2 (2.9) | * |
| Bone stress | 0 | | 2 (2.9) | * |
| Fracture | 2 (2.9) | * | 0 | |
| **Cartilage/Synovia/Bursa** | | | | |
| All | 4 (5.7) | * | 4 (5.7) | * |
| Cartilage | 1 (1.4) | * | 0 | |
| Synovitis/Capsulitis | 1 (1.4) | * | 3 (4.3) | * |
| Arthritis | 2 (2.9) | * | 1 (1.4) | |
| **Ligament/Joint Capsule** | | | | |
| All | 16 (22.9) | 18.80 (11.15–31.03) | 6 (8.6) | 0.19 (0.08–0.43) |
| Joint sprain | 16 (22.9) | 18.80 (11.15–31.03) | 6 (8.6) | 0.19 (0.08–0.43) |
| **Muscle/Tendon** | | | | |
| All | 6 (8.6) | 7.05 (2.87–16.06) | 18 (25.7) | 0.57 (0.35–0.91) |
| Muscle contusion | 2 (2.9) | * | 1 (1.4) | * |
| Muscle injury | 1 (1.4) | * | 2 (2.9) | * |
| Tendinopathy | 3 (4.3) | * | 15 (21.4) | 0.47 (0.27–0.80) |
| **Nervous** | | | | |
| All | 3 (4.3) | * | 3 (4.3) | * |
| Brain/Spinal cord injury | 2 (2.9) | * | 3 (4.3) | * |
| Peripheral nerve injury | 1 (1.4) | * | 0 | |
| **Superficial tissues/skin** | | | | |
| All | 3 (4.3) | * | 2 (2.9) | |
| Contusion (superficial) | 0 | | 2 (2.9) | * |
| Laceration | 1 (1.4) | * | 0 | * |
| Abrasion | 2 (2.9) | * | 0 | |
| **Vessels** | | | | |
| All | 1 (1.4) | * | 0 | |
| Vascular trauma | 1 (1.4) | * | 0 | |

n: number of injuries reported in the study. %: frequency of total injuries reported in the study.
I: incidence per 1000 player-hours. 95% CI: 95% Confidence Intervals.
*Number too small to calculate incidence. Incidence and 95%CI only reported for values $n \geq 5$

Supplementary Table 3: Match and training injuries by body region, body area, diagnosis and impact

| **Body Region**<br>**Body area**<br>**Clinical diagnosis** | **Match Injuries** | | | | | **Training Injuries** | | | | |
|---|---|---|---|---|---|---|---|---|---|---|
| | **n (%)** | **TL** | **RD** | **MA** | **ID** | **n (%)** | **TL** | **RD** | **MA** | **ID** |
| **Head & Neck** | **2 (2.9)** | **39** | **2** | **0** | **41** | **4 (5.7)** | **0** | **18** | **38** | **18** |
| **Head** | 2 (2.9) | 39 | 2 | 0 | 41 | 4 (5.7) | 0 | 18 | 38 | 18 |
| Brain/Spinal cord | 2 (2.9) | 39 | 2 | 0 | 41 | 3 (4.3) | 0 | 18 | 37 | 18 |
| Contusion | 0 | | | | | 1 (1.4) | 0 | 0 | 1 | 0 |
| **Upper Limb** | **3 (4.3)** | **52** | **0** | **259** | **52** | **3 (4.3)** | **0** | **2** | **149** | **2** |
| **Shoulder** | 1 (1.4) | 0 | 0 | 16 | 0 | 2 (2.9) | 0 | 2 | 56 | 2 |
| Synovitis/Capsulitis | 1 (1.4) | 0 | 0 | 16 | 0 | 1 (1.4) | 0 | 0 | 45 | 0 |
| Contusion | 0 | | | | | 1 (1.4) | 0 | 2 | 11 | 2 |
| **Forearm** | 1 (1.4) | 52 | 0 | 0 | 52 | 0 | | | | |
| Fracture | 1 (1.4) | 52 | 0 | 0 | 52 | 0 | | | | |
| **Hand** | 1 (1.4) | 0 | 0 | 243 | 0 | 1 (1.4) | 0 | 0 | 93 | 0 |
| Fracture | 1 (1.4) | 0 | 0 | 243 | 0 | 0 | | | | |
| Synovitis/Capsulitis | 0 | | | | | 1 (1.43) | 0 | 0 | 93 | 0 |
| **Trunk** | **3 (4.3)** | **0** | **2** | **38** | **2** | **3 (4.3)** | **5** | **3** | **60** | **8** |
| **Chest** | 0 | | | | | 1 (1.4) | 5 | 0 | 0 | 5 |
| Muscle injury | 0 | | | | | 1 (1.4) | 5 | 0 | 0 | 5 |
| **Thoracic spine** | 0 | | | | | 1 (1.4) | 0 | 0 | 2 | 0 |
| Joint sprain | 0 | | | | | 1 (1.4) | 0 | 0 | 2 | 0 |
| **Lumbar spine** | 3 (4.3) | 0 | 2 | 38 | 2 | 1 (1.4) | 0 | 3 | 58 | 3 |
| Nerve injury | 1 (1.4) | 0 | 0 | 31 | 0 | 0 | | | | |
| Arthritis | 2 (2.9) | 0 | 2 | 7 | 2 | 1 (1.4) | 0 | 3 | 58 | 3 |
| **Lower Limb** | **27 (38.6)** | **558** | **141** | **950** | **699** | **25 (35.7)** | **417** | **177** | **694** | **594** |
| **Hip/Groin** | 1 (1.4) | 0 | 0 | 1 | 0 | 0 | | | | |
| Abrasion | 1(1.4) | 0 | 0 | 1 | 0 | 0 | | | | |
| **Thigh** | 4 (5.7) | 0 | 4 | 73 | 4 | 1 (1.4) | 4 | 0 | 2 | 4 |
| Muscle injury | 1 (1.4) | 0 | 0 | 15 | 0 | 1 (1.4) | 4 | 0 | 2 | 4 |
| Muscle contusion | 2 (2.9) | 0 | 0 | 5 | 0 | 0 | | | | |
| Vascular trauma | 1 (1.4) | 0 | 0 | 53 | 0 | 0 | | | | |
| **Knee** | 4 (5.7) | 208 | 0 | 265 | 208 | 5 (7.1) | 288 | 57 | 27 | 345 |
| Laceration | 1 (1.4) | 0 | 0 | 7 | 0 | 0 | | | | |
| Tendinopathy | 1 (1.4) | 0 | 0 | 50 | 0 | 3 (4.3) | 0 | 57 | 27 | 57 |
| Cartilage injury | 1 (1.4) | 0 | 0 | 208 | 0 | 0 | | | | |
| Synovitis/Capsulitis | 0 | | | | | 1 (1.4) | 5 | 0 | 0 | 5 |
| Joint Sprain | 1 (1.4) | 208 | 0 | 0 | 208 | 1 (1.4) | 283 | 0 | 0 | 283 |
| **Lower leg** | 2 (2.9) | 0 | 3 | 338 | 3 | 10 (14.3) | 0 | 63 | 439 | 63 |
| Tendinopathy | 2 (2.9) | 0 | 3 | 338 | 3 | 10 (14.3) | 0 | 63 | 439 | 63 |
| **Ankle** | 15 (21.4) | 350 | 134 | 255 | 484 | 6 (8.6) | 125 | 49 | 176 | 174 |
| Joint Sprain | 15 (21.4) | 350 | 134 | 255 | 484 | 4 (5.7) | 125 | 49 | 104 | 174 |
| Tendinopathy | 0 | | | | | 1 (1.4) | 0 | 0 | 35 | 0 |
| Muscle contusion | 0 | | | | | 1 (1.4) | 0 | 0 | 37 | 0 |
| **Foot** | 1 (1.4) | 0 | 0 | 18 | 0 | 3 (4.3) | 0 | 8 | 50 | 8 |
| Bone | 0 | | | | | 2 (3.0) | 0 | 8 | 9 | 8 |
| Muscle/tendon | 0 | | | | | 1 (1.4) | 0 | 0 | 41 | 0 |
| Abrasion | 1 (1.4) | 0 | 0 | 18 | 0 | 0 | | | | |

n: number of injuries reported in the study. %: frequency of total injuries reported in the study.
TL: Time-loss days, RD: Restricted days, MA: Medical attention days, ID: Overall Impact Duration (Time-Loss days + Restricted days)
